# Emergence and Clonal Spread of CTX-M-65-Producing *Escherichia coli* From Retail Meat in Portugal

**DOI:** 10.3389/fmicb.2021.653595

**Published:** 2021-07-20

**Authors:** Célia Leão, Lurdes Clemente, Laura Moura, Anne Mette Seyfarth, Inge M. Hansen, Rene S. Hendriksen, Ana Amaro

**Affiliations:** ^1^Laboratory of Bacteriology and Mycology, National Institute of Agrarian and Veterinary Research (INIAV, IP), Oeiras, Portugal; ^2^MED – Mediterranean Institute for Agriculture, Environment and Development, Évora, Portugal; ^3^Faculty of Veterinary Science, CIISA- Centre for Interdisciplinary Research in Animal Health, Lisbon, Portugal; ^4^Faculty of Pharmacy, University of Lisbon, Lisbon, Portugal; ^5^EURL-AR, European Reference Laboratory for Antimicrobial Resistance, Technical University of Denmark (DTU), National Food Institute, Lyngby, Denmark

**Keywords:** *Escherichia coli*, retail meat, ESBL, WGS, CTX-M-65, chromosome

## Abstract

The emergence and dissemination of resistance to third- and fourth-generation cephalosporins among *Enterobacteriaceae* from different sources impose a global public health threat. Here, we characterized by whole-genome sequencing four *Escherichia coli* strains harboring the *bla*_CTX–M–65_ gene identified among 49 isolates from beef and pork collected at retail. The genomic content was determined using the Center for Genomic Epidemiology web tools. Additionally, the prediction and reconstruction of plasmids were conducted, the genetic platform of the *bla*_CTX–M–65_ genes was investigated, and phylogenetic analysis was carried out using 17 other genomes with the same sequence type and harboring the *bla*_CTX–M–65_ gene. All strains harbored *bla*_CTX–M–65_, *bla*_OXA–1_, and *bla*_TEM–1B_, and one also carried the *bla*_SHV–12_ gene. Other resistance genes, namely, *qnrS2*, *aac(6′)-Ib-c*, *dfrA14*, *sul2*, *tetA*, and *mphA*, were present in all the genomes; the *mcr*-1.1 gene was identified in the colistin-resistant strains. They belong to sequence type 2179, phylogenetic group B1, and serotype O9:H9 and carried plasmids IncI, IncFIC(FII), and IncFIB. All strains share an identical genetic environment with IS*903* and IS*Ecp1* flanking the *bla*_CTX–M–65_ gene. It seems likely that the *bla*_CTX–M–65_ gene is located in the chromosome in all isolates based on deep *in silico* analysis. Our findings showed that the strains are clonally related and belong to two sub-lineages. This study reports the emergence of CTX-M-65-producing *E. coli* in Portugal in food products of animal origin. The chromosomal location of the *bla*_CTX–M–65_ gene may ensure a stable spread of resistance in the absence of selective pressure.

## Introduction

Bacteria harboring antimicrobial resistance genes can be spread to humans, constituting a global public health concern (O’Neill, 2016). Of particular importance is the resistance to β-lactam antibiotics, enzymatic inactivation being the most common mechanism of resistance through which β-lactamases cause the cleavage of the β-lactam ring (Blair et al., 2015). Extended-spectrum β-lactamase (ESBL) is a group of enzymes that hydrolyze oxymino-beta-lactam antibiotics, conferring resistance to a wide variety of β-lactams, including penicillins, first-, second-, third-, and fourth-generation cephalosporins, and monobactams (Bush, 2018). These enzymes can be inhibited by β-lactam inhibitors such as clavulanic acid, sulbactam, and tazobactam through the covalent link to the serine residue active site (Tooke et al., 2019). Mobile genetic elements, particularly plasmids, are involved in the spread of ESBL genes, resulting in the rapid increase of ESBL-producing bacteria among different sources (Rozwandowicz et al., 2018; Silva et al., 2019). ESBLs are classified into several enzymatic groups, of which CTX-M, OXA, SHV, and TEM are frequently observed, CTX-M being the most prevalent (Silva et al., 2019; Palmeira and Ferreira, 2020). Currently, 230 CTX-M variants have been described according to GenBank records (last accessed on January 4, 2021). In Europe, the most frequently reported variants of *Enterobacteriaceae* species isolated from food-producing animals and food products are CTX-M-1, CTX-M-14, CTX-M-15, and CTX-M-2, CTX-M-15 being associated with outbreaks of severe extraintestinal infections in humans caused by the multidrug-resistant (MDR) *Escherichia coli* ST131 (Silva et al., 2019; Palmeira and Ferreira, 2020). In Portugal, the CTX-M-1, CTX-M-14, and CTX-M-32 variants were reported as the most prevalent in commensal and pathogenic *E. coli* isolated from food-producing animals and food products (Clemente et al., 2019; Silva et al., 2019).

In 2008, a new variant of the CTX-M family identified as CTX-M-65, belonging to the CTX-M-9 group and cluster 14, was described for the first time in *E. coli* isolated from a human urine sample in the United States (Doi et al., 2008). CTX-M-65 differs from CTX-M-14 by two amino acid substitutions, namely, alanine by valine at position 77 (A77V) and serine by arginine at position 274 (S274R). In 2017, Tate and colleagues reported the emergence of a CTX-M-65 *Salmonella* Infantis isolated from food animals, retail chickens, and humans in the United States that is highly resistant to most of the studied β-lactam antimicrobials (Tate et al., 2017). In Europe, the CTX-M-65 variant has also been reported from Italy in *Salmonella* Infantis isolated from broilers and humans (Franco et al., 2015) and from the Netherlands in *E. coli* from cattle (Palmeira and Ferreira, 2020). Globally, the enzyme is widely distributed, with reports from China, Korea, and South America in *E. coli* isolated from humans, food-producing animals, retail chickens, and in a giant anteater from a zoo (Zheng et al., 2012; Bartoloni et al., 2013; Rao et al., 2014; Tate et al., 2017; Furlan et al., 2019; Park et al., 2019; Vinueza-Burgos et al., 2019; Wang et al., 2020). It is noteworthy to mention that a multidrug carbapenemase strain of *Klebsiella pneumoniae* co-producing CTX-M-65 was the causative agent of a severe nosocomial outbreak in China (Zhan et al., 2017).

In the present study, four CTX-M-65-producing *E. coli* isolated from beef and pork samples collected at retail in 2017 were characterized by whole-genome sequencing (WGS). Here, we described the molecular epidemiology of the resistance genes, the identification of the plasmids, and the *bla*_CTX–M–65_ genetic environment. Moreover, we determined and described the genetic relatedness with other *E. coli* genomes for better insights into the public health impact of an ESBL producer rarely found in Europe.

## Materials and Methods

### Bacterial Isolates and Antimicrobial Susceptibility Testing

Two hundred and twenty beef and 220 pork samples were collected at retail stores across mainland Portugal in compliance with the European Commission Implementing Decision of November 12, 2013 to monitor and report antimicrobial resistance in zoonotic and commensal bacteria (Commission Decision 652/2013) in 2017. The isolation and identification of extended-spectrum β-lactamase/plasmid-mediated AmpC (ESBL/PMAβ) *E. coli* producers from meat samples were performed according to the laboratory protocols defined by the European Union Reference Laboratory for antimicrobial resistance (EURL-AR)^1^. Briefly, 25 g of each meat sample was mixed with 225 ml of buffered peptone water, followed by incubation at 37°C for 18–22 h. Enriched samples were plated onto MacConkey agar supplemented with 1 mg/L of cefotaxime (Glentham, Corsham, United Kingdom) and incubated at 44°C for 18–22 h. Presumptive *E. coli* colonies were selected for biochemical identification on ChromID^®^ coli agar (bioMérieux, Marcy-l’Étoile, France), and after confirmation, the isolates were sub-cultured and stored at −80°C before further analyses.

The isolates were tested for antimicrobial susceptibility through the determination of the minimum inhibitory concentrations (MICs) using commercially available 96-well microplates, EUVSEC and EUVSEC2 (Sensititre^®^, Trek Diagnostic Systems, East Grinstead, United Kingdom) panels, and the results were interpreted according to EUCAST epidemiological breakpoints^2^.

### Molecular Characterization of β-Lactam Resistance

Resistance mechanisms associated with ESBL/PMAβ enzymes were screened by PCR using primers targeting *bla*_TEM_, *bla*_SHV_, *bla*_OXA_, *bla*_CTX–M_, *bla*_ACC_, *bla*_FOX_, *bla*_MOX_, *bla*_DHA_, *bla*_CIT_, and *bla*_EBC_ (Dallenne et al., 2010). Amplified products were purified with ExoSAP-IT^TM^ (Applied Biosystems^TM^, Warrington, United Kingdom), followed by Sanger sequencing using the BigDye^®^ Terminator v3.1 Cycle Sequencing Kit (Applied Biosystems). The sequencing of fragments was performed in an automatic sequencer ABI3100 (Applied Biosystems), and the identification of resistance genes was determined using the Basic Local Alignment Search Tool (BLAST) from the NCBI website (Altschul et al., 1990) and The Comprehensive Antibiotic Resistance Database (CARD) (Alcock et al., 2019).

### Whole-Genome Sequencing and Bioinformatics Analysis of the CTX-M-65 *E. coli* Producers

Four isolates identified as positive for *bla*_CTX–M–65_ by PCR and Sanger sequencing were further characterized by whole-genome sequencing. The isolates were recovered from pork and beef samples, collected on the same day from the same retail store in the north of Portugal (INIAV_ECX027 and INIAV_ECX036) and on different days from different retail stores in Lisbon and Tejo Valley (INIAV_ECX016 and INIAV_ECX035).

The genome of two isolates (INIAV_016ECX and INIAV_027ECX) was sequenced at EURL-AR, DTU, Lyngby, Denmark, under the scope of the European Food Safety Authority (EFSA) confirmatory testing. Genomic DNA was extracted using an Invitrogen Easy-DNA Kit^TM^ (Invitrogen, Carlsbad, CA, United States) and the DNA concentrations determined using the Qubit dsDNA BR assay kit (Invitrogen). Genomic DNA was prepared for Illumina pair-end sequencing using the Illumina (Illumina, Inc., San Diego, CA, United States) Nextera XT^®^ Guide following the protocol revision C1. A sample of the pooled Nextera XT Libraries was loaded onto an Illumina MiSeq reagent cartridge using MiSeq Reagent Kit v3. The libraries were sequenced using an Illumina MiSeq platform (Illumina). The raw reads were *de novo* assembled using the assembler pipeline (version 1.4) available from the Center for Genomic Epidemiology (CGE)^3^. Raw sequence data from these two isolates were submitted to the European Nucleotide Archive (ENA)^4^ under study accession numbers: ERS3535656 and ERS3535669.

DNA extraction of the remaining two isolates (INIAV_035ECX and INIAV_036ECX) was carried out at INIAV, Oeiras, Portugal, using the PureLink^®^ Genomic DNA kit (Invitrogen) according to the manufacturer’s instructions, with minor modifications. Briefly, the incubation period at 55°C was performed for 90 min and the DNA eluted with 50 μl of Tris-HCl buffer, pH 8.5. The DNA quality and quantity were assessed using a spectrophotometer [NanoDrop^®^ 2000, Thermo Scientific, emergency use authorization (EUA), Waltham, MA, United States] and sequenced using the Illumina HiSeq sequencing technology (NovaSeq 6000 S2 PE150 XP sequencing mode, Eurofins Genomics Europe Sequencing GmbH, Ebersberg, Germany). The raw sequence data from these two isolates were submitted to the ENA under study accession numbers: ERS5493675 and ERS5493676. Raw data quality was assessed by FastQC^5^. BBDuk from the BBTools package^6^ was used to remove possible contamination by adapter sequences and for trimming/removing low-quality reads, all performed with a minimum quality of Q20 using the Phred algorithm, with a minimum read length of 50 and with a *k*-mer length parameter of 19. All pre-processed reads were assembled with SPAdes 3.12.0 (Nurk et al., 2013).

The assembly stats of all the sequenced isolates were calculated using QUAST-5.0.2 (Mikheenko et al., 2018). Contigs with sizes lower than 500 bp were removed, and bioinformatics analysis using tools available at the CGE was performed. The acquired antimicrobial resistance genes and chromosomal point mutations, plasmid replicons, multilocus sequence type (MLST), serotype, *fum*C and *fim*H type, identification of virulence genes, and pathogenicity were assessed using ResFinder version 4.1 (80% threshold for%ID/60% minimum length) (Zankari et al., 2012; Bortolaia et al., 2020), PlasmidFinder version 2.1 (80% threshold for%ID) (Carattoli et al., 2014), MLST version 2.0 (Larsen et al., 2012), SerotypeFinder version 2.0 (85% threshold for%ID/60% minimum length) (Joensen et al., 2015), CHTyper version 1.0 (95% threshold for%ID) (Camacho et al., 2009), VirulenceFinder version 2.0 (90% threshold for%ID/60% minimum length) (Joensen et al., 2014), and PathogenFinder version 1.1 (Cosentino et al., 2013), respectively. The phylogenetic group was predicted using the ClermonTyping web-based tool (Beghain et al., 2018; Clermont et al., 2019).

Additionally, PLACNETw (Plasmid Constellation Network) was used to predict and reconstruct the plasmids (Vielva et al., 2017) in specifically assembled contigs of the bacterial genomes. For identifying the genetic platform of the CTX-M-65 enzyme, the contigs containing the *bla*_CTX–M–65_ gene were annotated using Prokka version 1.14.6 (Seemann, 2014), followed by analysis with Artemis (Carver et al., 2012) and EasyFig version 2.2.5 (Sullivan et al., 2011). A plasmid database, PLSDB, was also used to search for the plasmid nucleotide sequences contained in each of the selected contigs using the “mash screen” option (Galata et al., 2018).

A phylogenetic analysis based on the single nucleotide polymorphisms (SNPs) present in the genomes using CSI Phylogeny version 1.4 (10 reads of minimal depth at SNP positions, 10% minimal relative depth at SNP positions, 10 bp of minimal distance between SNPs, minimal SNP quality of 30, minimal read mapping quality of 25, and a minimal *Z*-score of 1.96) (Kaas et al., 2014) from the CGE website was conducted with the *E. coli* isolates from this study and 17 *E. coli* genomes with the same sequence type (ST2179) and harboring the *bla*_CTX–M–65_ gene, retrieved from EnteroBase (Zhou et al., 2020). The tree was visualized using FigTree version v1.4.3^7^.

## Results

### Characterization of Antimicrobial Resistance

From 49 isolates of *E. coli* phenotypically resistant to third-generation cephalosporins, a total of 10 different *bla* genes were identified in 42 ESBL and seven PMAβ producers: *bla*_CTX–M–1_ (*n* = 8), *bla*_CTX–M–15_ (*n* = 5), *bla*_CTX–M–27_ (*n* = 2), *bla*_CTX–M–55_ (*n* = 1), *bla*_CTX–M–9_ (*n* = 1), *bla*_CTX–M–14_ (*n* = 7), *bla*_CTX–M–32_ (*n* = 9), *bla*_CTX–M–65_ (*n* = 4), *bla*_SHV–12_ (*n* = 5), and *bla*_CMY–2_ (*n* = 7).

The *bla*_CTX–M–65_ gene was detected in four isolates (8.2%) recovered from beef (3/26) and pork (1/23) retail meat samples, and all exhibited a MDR phenotype, being resistant to ciprofloxacin, nalidixic acid, azithromycin, chloramphenicol, tetracycline, trimethoprim, and sulfamethoxazole. Resistance to colistin was observed in all, except isolate INIAV_ECX035 (Table 1).

**TABLE 1:** Results of the minimum inhibitory concentrations (μg/ml) obtained by antimicrobial susceptibility testing.

**Antibiotic**	**Breakpoints**	**INIAV_ECX016**	**INIAV_ECX027**	**INIAV_ECX035**	**INIAV_ECX036**
Ampicillin	8	>64	>64	>64	>64
Cefepime	0.125	4	2	4	8
Cefotaxime	0.25	64	64	64	>64
Cefoxitin	8	8	8	8	8
Ceftazidime	0.5	1	1	32	2
Ciprofloxacin	0.064	>8	>8	>8	>8
Nalidixic acid	16	>128	>128	>128	>128
Colistin	2	4	4	≤1	4
Ertapenem	0.064	≤0.015	≤0.015	≤0.015	≤0.015
Imipenem	0.5	0.25	0.25	0.25	0.5
Meropenem	0.125	≤0.03	≤0.03	≤0.03	≤0.03
Tetracycline	8	64	>64	64	>64
Sulfamethoxazole	64	>1,024	>1,024	>1,024	>1,024
Trimethoprim	2	>32	>32	>32	>32
Chloramphenicol	16	128	128	128	128
Gentamicin	2	1	1	1	2
Azithromycin	16	32	32	64	64
Tigecycline	1	0.5	0.5	0.5	0.5
Temocillin	32	8	8	8	8
Cefotaxime/clavulanic acid	0.25	≤0.06	≤0.06	0.12	0.12
Ceftazidime/clavulanic acid	0.5	≤0.12	0.25	≤0.12	0.25

### Genome Analysis of the CTX-M-65 *E. coli* Producers

The assemblies of the reads originated between 70 and 120 contigs and a genome size of about 5 Gb. The genotypic traits of the four isolates, their resistome and mobilome, are summarized in Table 2. According to the ResFinder tool, three β-lactam-encoding genes were found in all isolates, namely, *bla*_CTX–M–65_, *bla*_OXA–1_, and *bla*_TEM–1B_. Moreover, one isolate (INIAV_ECX035) also carried *bla*_SHV–12_. The *mcr*-1.1 gene was identified in the three isolates resistant to colistin, and plasmid-mediated quinolone resistance (PMQR) genes, namely, *qnrS2* and *aac(6′)-Ib-cr*, were detected in all isolates. Additional genes conferring resistance to trimethoprim/sulfamethoxazole (*dfrA14* and *sul2*), tetracycline (*tetA*), and azithromycin (*mphA*) were also present. Point mutations were identified at the *gyrA* and *parC* subunits of DNA, namely, serine by lysine at position 83 (S83L) and serine by isoleucine at position 80 (S80I), respectively, conferring resistance to quinolones. Based on these results, the phenotype and genotype were in accordance.

**TABLE 2:** Genomic characterization of the four CTX-M-65-producing *E. coli* isolates by whole-genome sequencing.

**Features**		**INIAV_ECX016**	**INIAV_ECX027**	**INIAV_ECX035**	**INIAV_ECX036**
Antibiotic resistance determinants	Ampicillin, Cefepime, Cefotaxime, Cefoxitin, Ceftazidime	*bla*_CTX–M–65_, *bla*_TEM–1B_, *bla*_OXA–1_	*bla*_CTX–M–65_, *bla*_TEM–1B_, *bla*_OXA–1_	*bla*_CTX–M–65_, *bla*_TEM–1B_, *bla*_OXA–1_, *bla*_SHV–12_	*bla*_CTX–M–65_, *bla*_TEM–1B_, *bla*_OXA–1_
	Ciprofloxacin, Nalidixic acid	*QnrS2*, *aac(6’)Ib-cr*, *gyrA* S83L, *parC* S80I	*QnrS2*, *aac(6’)Ib-cr*, *gyrA* S83L, *parC* S80I	*QnrS2*, *aac(6’)Ib-cr*, *gyrA* S83L, *parC* S80I	*QnrS2*, *aac(6’)Ib-cr*, *gyrA* S83L, *parC* S80I
	Colistin	*mcr*-1.1	*mcr*-1.1	–	*mcr*-1.1
	Tetracycline	*tet(A)*	*tet(A)*	*tet(A)*	*tet(A)*
	Sulphamethoxazole	*sul2*	*sul2*	*sul2*, *sul1*	*sul2*
	Trimethoprim	*dfrA14*	*dfrA14*	*dfrA17*	*dfrA14*
	Chloramphenicol	*catB3*, *floR*	*catB3*, *floR*	*catB3*, *floR*	*catB3*, *floR*
	Azithromycin	*mph(A)*	*mph(A)*	*mph(A)*	*mph(A)*
	Rifampicin	*ARR-3*	*ARR-3*	*ARR-3*	*ARR-3*
	Aminoglycoside	*aph(3*″*)-Ib*, *aph(6)-Id*	*aph(3*″*)-Ib*, *aph(6)-Id*	*aadA5*, *aph(3*″*)-Ib*, *aph(6)-Id*	*aph(3*″*)-Ib*, *aph(6)-Id*
	Other	*mdf(A)*	*mdf(A)*	*mdf(A)*	*mdf(A)*
Plasmid replicons		IncI2, pIncFIB, p0111, IncFIC	IncI2, pIncFIB, IncFIC	IncI1-I, IncFIB, IncFIC (FII)	IncI2, IncFIB, IncFIC(FII)
MLST		ST2179	ST2179	ST2179	ST2179
Serotype		O9:H9	O9:H9	O9:H9	H9
Virulence genes		*lpfA*, *iroN*, *iss*, *cma*	*lpfA*, *iroN*, *iss*, *cma*, *gad*	*lpfA*, *iroN*, *iss*, *cma*, *gad*	*lpfA*, *iroN*, *iss*, *cma*, *gad*
*fum*C/*fim*H type		*fum*C65/*fim*H32	*fum*C65/*fim*H32	*fum*C65/*fim*H32	*fum*C65/*fim*H32
Pathogenicity		Yes (93.6%)	Yes (93.6%)	Yes (93.3%)	Yes (93.6%)
Phylogenetic group		B1	B1	B1	B1
Sample source		Bovine	Swine	Bovine	Bovine
No. of contigs		90	77	74	79
Total length of genome (bp)		5,115,923	5,012,767	5,015,983	5,058,472
N50 (bp)		140,276	142,205	282,032	232,144
					

According to the PlasmidFinder tool, IncFIC (FII) and IncFIB replicons were identified in all isolates, and p0111 was also predicted in one isolate (INIAV_ECX016). Isolate INIAV_ECX035 also harbored the IncI1-I plasmid, while the remaining isolates carried the IncI2 plasmid. All strains have identical profiles regarding MLST (ST2179), *fum*C65/*fim*H32 alleles, serotype (O9:H9), and phylogroup (B1). The somatic antigen of the INIAV_ECX036 isolate was not typable. VirulenceFinder predicted the presence of five virulence factors (*lpfA*, *iroN*, *iss*, *cma*, and *gad*) in all isolates except one (INIAV_ECX016), which carries four as *gad* is absent.

**FIGURE 1:**
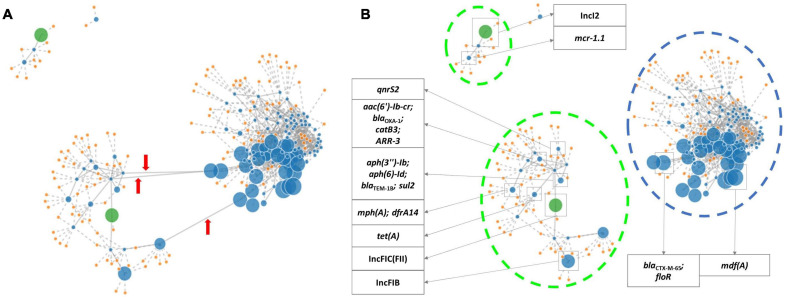
PLACNETw (Plasmid Constellation Network) representation of the INIAV_ECX036 genome. **(A)** The original network. *Red arrows* indicate the contig linkages that were pruned. **(B)** The pruned network, where contigs containing the antimicrobial resistance genes and plasmids are identified according to ResFinder and PlasmidFinder. *Blue dots* indicate contigs (the dot size is proportional to the size of the contig); *orange dots* are the reference genomes from the tool database; *green dots* are contigs with relaxase and replicon protein of the plasmid; *blue broken lines* indicate contigs identified as chromosome; and *green broken lines* are contigs identified as plasmids.

Using PLACNETw, a new assembly was generated from the raw reads, and a network was produced representing the contigs defined as belonging to the chromosome and plasmids, according to the reference genomes from the software database. The contigs were identified as belonging to plasmids based on the recognition of relaxase and/or replicon protein sequences. A manual pruning of the original network (Figure 1A) was performed according to Lanza et al. (2014) to reconstruct the graphical representation of the genome (Figure 1B). With the FASTA files obtained from this assembly, a new analysis with PlasmidFinder and ResFinder was performed to identify in which contigs were the antimicrobial resistance genes and plasmids located. Hereafter, it was possible to pinpoint the exact position of the resistance genes in the genome’s graphical representation. Thus, based on the analysis of all isolates, the *bla*_CTX–M–65_ gene, along with *flo*R and *mdf*A, was identified in contigs belonging to the chromosome, and the remaining resistance genes, including the other *bla* genes, were located in contigs belonging to plasmids (Figure 1B). In isolates INIAV_ECX016, INIAV_ECX027, and INIAV_ECX036, genes *bla*_CTX–M–65_ and *flo*R were located in the same contig, and *mdf*A was in a different contig, while in isolate INIAV035, the three genes were in different contigs. Most resistance genes were located in IncFIC (FII) and IncFIB plasmids, except *bla*_SHV–12_ and *mcr*1.1, which were carried in IncI-1 and IncI2, respectively.

**FIGURE 2:**
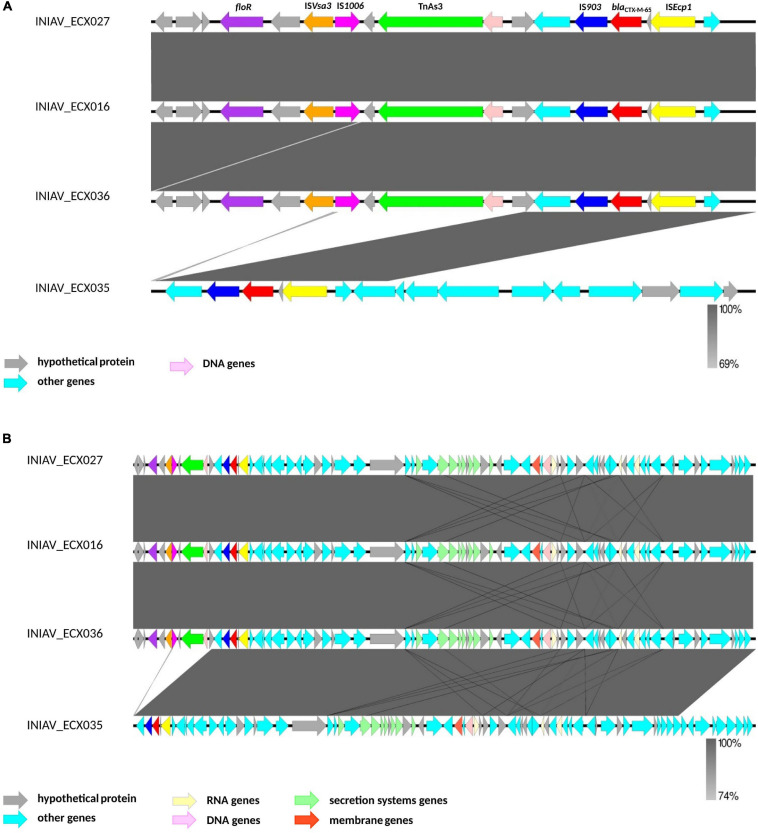
Genetic platform of the *bla_CTX–M–65_* genes obtained by the analysis of the contigs containing the *bla*_*CTX*_ gene using EasyFig. **(A)** A subregion of 17,000 bp of each contig to better identify the mobilization elements. **(B)** A subregion of 83,000 bp from the contigs.

The analysis of the genetic platform of the *bla*_CTX–M–65_ genes using the EasyFig tool revealed that all isolates have the IS*903* (IS*5* family) and IS*Ecp1* (IS*1380* family) flanking the *bla*_CTX–M–65_ gene, and all but one isolate also harbored the transposon TnAs3 (Tn3 family), IS*1006* (IS6 family) and IS*Vsa3* (IS*91* family). In this isolate (INIAV_ECX035), the contig starts in a different position, and its genetic platform seems to be not fully represented (Figure 2A). Consequently, the genetic platform is split and the downstream elements (TnAs3, IS*1006*, and IS*Vsa3*) are not represented in this contig, being in a different one. Looking at a larger region of the contigs (Figure 2B), it is possible to realize that all four contigs have high homology between each other. The alignment of each contig using EasyFig with the plasmid sequences identified by PlasmidFinder revealed no homology between those sequences. Comparison of the sizes of the contigs containing the *bla*_CTX–M–65_ gene (ranging from 227,681 to 340,574 bp, except for the INIAV_ECX035 isolate with 82,631 bp in length) with the sizes of the plasmids identified in this study (ranging from 64,015 to 99,159 bp) revealed that the contigs are mostly wider than the plasmids. Furthermore, no plasmids were also identified, using the “mash screen” option of the PLSDB tool, in the contig’s input sequence containing the *bla*_CTX–M–65_ gene from each isolate.

**FIGURE 3:**
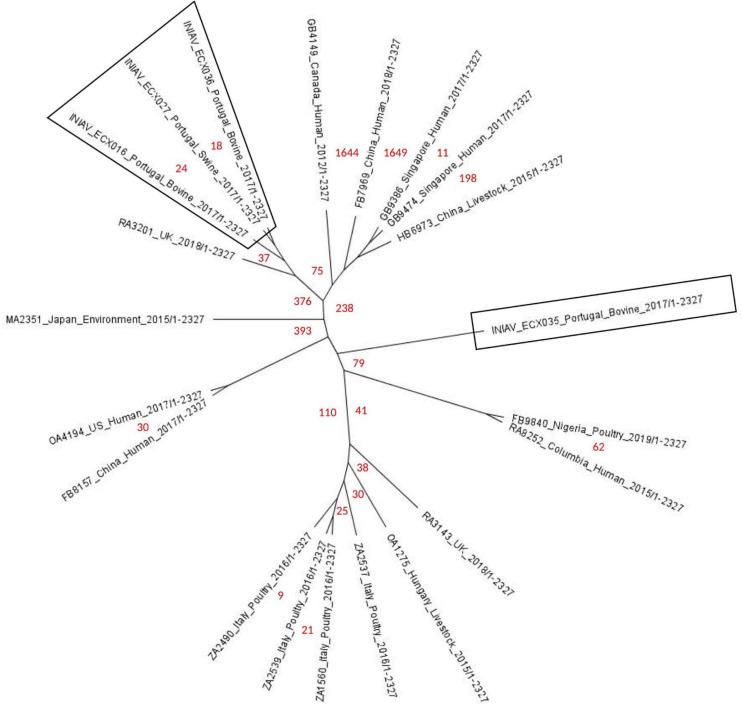
Phylogenetic tree of the 21 ST2179 *Escherichia coli* strains harboring the *bla*_CTX–M–65_ gene generated by single nucleotide polymorphism (SNP) analysis using the CSIPhylogeny tool and visualized with FigTree (unrooted radial cladogram options). *Numbers in red* represent the number of SNPs between the strains from distinct geographic regions and sources. Portuguese strains are *inside boxes*.

An unrooted phylogenetic tree (radial cladogram option) using *E. coli* ATCC 25922 as the reference genome was constructed using 21 genomes from different geographic regions worldwide and isolated from multiple sources (Figure 3). Multiple phylogenetic groups can be distinguished in the tree: one group includes two strains, one from Nigeria and one from Colombia isolated from poultry and human samples; another phylogenetic group including European strains from the United Kingdom, Hungary, and Italy, isolated mainly from poultry; other groups formed with one strain from the United States and one strain from China isolated from human samples; another group includes strains from China, Singapore, and Canada isolated from human and livestock samples; and two groups with only one strain each from Portugal and Japan isolated from bovine and environmental samples. All shared about 43,430 SNPs against the reference genome. The SNP analysis showed FB7969_China_Human_2018 as the strain having more SNPs, between 1,636 and 1,746. Most shared less than 112 SNPs with the four strains from this study, except for HB6973_China_Livestock_2015 and MA2351_Japan_Environment_2015 separated by between 219 and 394 SNPs. Three Portuguese strains were grouped with one strain from the UK (RA3201_UK_2018), separated by 37 and 45 SNPs. The fourth Portuguese strain (INIAV_ECX035) is separated from the remaining three from this study by 85 and 101 SNPs, being in a different phylogenetic group. The most closely related strains from this study are INIAV_ECX016 and INIAV_ECX036, sharing only 8 SNPs between each other; these two strains were obtained from beef samples collected from distinct geographic regions in Portugal.

## Discussion

In this study, four CTX-M-65-producing *E. coli* were identified among 49 ESBL/PMAβ producers isolated from meat. Here, *bla*_CTX–M–65_ is identified in Portugal for the first time in food of animal origin, with no reports linking to human infections; a complete characterization by WGS of the four MDR *E. coli* harboring this gene is described. *E. coli* harboring the *bla*_CTX–M–65_ gene is commonly found in food-producing animals and meat from Southwest Asian and South American countries (Rao et al., 2014; Na et al., 2019; Vinueza-Burgos et al., 2019; Zurita et al., 2019). Although rarely occurring in Europe, previous studies have reported CTX-M-65-producing *E. coli* from Dutch beef calves (Ceccarelli et al., 2019) and wild birds from Switzerland (Zurfluh et al., 2019). In our study, three isolates were from beef and one from pork; therefore, we cannot confirm the source of the CTX-M-65 *E. coli* detected in meat. There are several potential sources of bacteria in meat, including the animals from which the meat was derived, cross-contamination from other products, equipment and the environment, and the workers who are producing and handling the meat (EFSA and ECDC, 2019).

Based on PLACNETw, the *bla*_CTX–M–65_ genes were located in contigs with homology to the chromosome. Moreover, the contigs containing this gene are wider than the plasmids identified in this study, and none of the four contigs showed homology to any sequence of the identified plasmids, reinforcing that the *bla*_CTX–M–65_ gene is in the chromosome. The chromosomal location of *bla*_CTX–M_ genes has already been reported, namely, *bla*_CTX–M–2_ (Zhao and Hu, 2013; Ferreira et al., 2014), *bla*_CTX–M–14_ and *bla*_CTX–M–15_ (Hamamoto and Hirai, 2019), *bla*_CTX–M–55_ (Zhang et al., 2019), and *bla*_CTX–M–65_ (He et al., 2017).

The successful spread of ESBL enzymes is based on their ability to disseminate their resistance genes on mobile genetic elements to other bacteria, the skill to expand their spectrum of activity, and the acquisition of point mutations (Bush, 2018; Galal et al., 2018; Tooke et al., 2019). Nevertheless, the chromosomal location of the resistance genes can benefit the stable propagation of resistance, regardless of the bacterial host’s habitat (Yoon et al., 2020). The genetic platform analysis revealed that all share an identical genetic environment with IS*903* and IS*Ecp1* flanking the *bla*_CTX–M–65_ gene downstream and upstream, respectively. IS*Ecp1* is one of the most important insertion sequences associated with *bla*_CTX–M_ genes (Canton et al., 2012; Zhao and Hu, 2013) and Tn3, a conjugative transposon. These mobile genetic elements found in the chromosome may promote excision and reintegration in a new chromosome or transference to other bacteria through a conjugative plasmid (Canton et al., 2012).

Particularly worrying is the co-occurrence of genes encoding resistance to other critically important antimicrobials, namely, fluoroquinolones, macrolides, and polymyxins, except for isolate INIAV_ECX035, which was susceptible to colistin. This isolate also carried the *bla*_SHV–12_ gene, exhibiting a higher MIC to ceftazidime (MIC = 32 μg/ml) compared to the remaining isolates (MIC = 1–2 μg/ml), which is in accordance with the previously described (Maina et al., 2011).

Of note is that all isolates were resistant to azithromycin and harbored the *mph(A)* gene, confirming this gene’s relevant role in macrolide susceptibility. As previously reported (Gomes et al., 2019), most of the *mph(A)*-carrying isolates show a MIC > 32 μg/L, as observed in our study, where the MIC values were between 32 and 64 μg/L. Moreover, other antimicrobial resistance genetic determinants were found in the isolates, including those also associated with resistance to antimicrobials frequently used in the rearing of food-producing animals, such as sulfamethoxazole (*sul1* and *sul2*), trimethoprim (*dfrA14* and *dfrA17*), phenicols (*catB3* and *floR*), tetracycline (*tetA*), and aminoglycosides [*aph*(3″)-Ib and *aph*(6)-Id] (ESVAC, 2020).

In all isolates, different replicon-typing plasmids were identified [IncFIC(FII) and IncFIB] carrying most of the resistance genes. IncF plasmids are frequently described from human and animal sources and are considered epidemic resistance plasmids, bearing the greatest variety of resistance genes in Enterobacteriaceae (Rozwandowicz et al., 2018). Although the precise gene location on plasmids was not determined in this study, based on PLACNETw, *mcr*-1.1 was located on IncI2 replicon-typing plasmid. IncI2 plasmids have been associated with the mobilization of *mcr* genes widely spread in Europe in *E. coli* isolates from animals and humans (Rozwandowicz et al., 2018; Migura-Garcia et al., 2020). The IncI-1 plasmid predominantly described in Europe was identified in the INIAV_ECX035 strain carrying *bla*_SHV–12_, in accordance with previous reports (Rozwandowicz et al., 2018).

WGS analysis also revealed that all isolates belong to ST2179, phylogroup B1, and *fumC*65/*fimH*32 type, suggesting that all have a common clonal origin, with minor differences. Recently, *E. coli* ST2179 bearing the *bla*_CTX–M–65_ gene but belonging to phylogroup A was reported from ducks in South Korea (Na et al., 2019). Isolates belonging to phylogroup B1 are commonly associated with non-pathogenic commensal *E. coli* reported from humans, animals, and food products (Bailey et al., 2010; Coura et al., 2015; Scheinberg et al., 2017; Belaynehe et al., 2018; Zurita et al., 2019).

The phylogenetic analysis revealed three isolates (INIAV_ECX016, INIAV_ECX027, and INIAV_ECX036) grouped in the same cluster, showing high genetic homology between each other. The UK strain (RA3201_UK_2018) was closely related to the Portuguese strains with 37–45 SNPs. The *in silico* analysis of the UK strain revealed the *bla*_CTX–M–65_ gene to also be located in the chromosome. These findings suggest the clonal spread of CTX-M-65-producing *E. coli* isolates in Europe. INIAV_ECX035 is separated from INIAV_ECX027 by 101 SNPs and from the remaining two strains by 85 SNPs. Although from different animal species, the isolates from the same retail store (INIAV_ECX027 and INIAV_ECX036) are closely related, pointing out the hypothesis of cross-contamination. Moreover, the phylogenetic tree and the WGS analysis suggest that the four isolates belong to two sub-lineages, one composed of the three strains that grouped and the second with the fourth strain. INIAV_ECX035 showed some differences regarding the resistance genes and plasmids compared with the other three strains: the presence of the *bla*_SHV–12_ gene conferring a higher resistance to ceftazidime and the absence of resistance to colistin, also the presence of the IncI1-I plasmid instead of the IncI2 plasmid carrying the *mcr*-1.1 gene. These genetic differences may justify the higher number of SNPs found and the existence of two evolutionary sub-lineages suggesting that the CTX-M-65 variant is emerging in our country.

The emergence and clonal spread of *E. coli* harboring *bla*_CTX–M_-_65_ can be a problem for the livestock industry and human health, given their multidrug resistance profile to critically important antimicrobials and their presence in the food supply chain. To our knowledge, this is the first time that CTX-M-65 is identified in Portugal in food products of animal origin. The chromosomal addition of the *bla*_CTX–M–65_ gene may ensure the spread of resistance in the absence of selective pressure. A better understanding of the factors that contribute to the emergence and dissemination of ESBL genes rarely seen in Europe, but highly prevalent in Southwest Asian and South American countries, is strongly advisable and is worthy of close monitoring. Tourism and migration flow to Portugal and the trade treaties established between countries by importing meat and meat products may be sources of cross-contamination with uncommon MDR strains, facilitating their dissemination to the community.

## Data Availability Statement

The datasets presented in this study can be found in online repositories. The names of the repository/repositories and accession number(s) can be found below. https://www.ebi.ac.uk/ ERS3535656, ERS3535669, ERS5493675, and ERS5493676.

## Author Contributions

CL contributed to the whole-genome sequencing (WGS) experiments, bioinformatics analysis, and interpretation of the data and wrote the manuscript. LM contributed to the laboratory experiments. AS, IH, and RH contributed to the WGS and bioinformatics analysis. LC and AA designed the study, interpreted the data, and reviewed and edited the manuscript. All authors read and approved the manuscript.

## Conflict of Interest

The authors declare that the research was conducted in the absence of any commercial or financial relationships that could be construed as a potential conflict of interest.

## Footnotes

1 https://www.eurl-ar.eu/protocols.aspx

2 https://mic.eucast.org/Eucast2/

3 http://cge.cbs.dtu.dk/services/all.php

4 http://www.ebi.ac.uk/ena

5 http://www.bioinformatics.babraham.ac.uk/projects/fastqc

6 http://jgi.doe.gov/data-and-tools/bb-tools/

7 http://tree.bio.ed.ac.uk/software/figtree/
